# Pharmaceutical and nutraceutical potential of natural bioactive pigment: astaxanthin

**DOI:** 10.1007/s13659-022-00347-y

**Published:** 2022-07-07

**Authors:** Apurva D. Patil, Pramod J. Kasabe, Padma B. Dandge

**Affiliations:** 1 Department of Biochemistry, Shivaji University, Kolhapur, 416004 Maharashtra India; 2 School of Nanoscience and Biotechnology, Shivaji University, Kolhapur, Maharashtra India

**Keywords:** Astaxanthin, Nutritional supplement, Therapeutic compound, Chronic diseases, Fertility enhancer

## Abstract

Astaxanthin (3,3′-dihydroxy-β,β-carotene-4,4′-dione) is an orange-red, lipophilic keto-carotenoid pigment. It is majorly found in marine ecosystems particularly in aquatic animals such as salmon, shrimp, trout, krill, crayfish, and so on. It is also synthesized in microalgae *Heamatococcus pluvialis*, *Chlorococcum*, *Chlorella zofingiensis*, red yeast *Phaffia rhodozyma* and bacterium *Paracoccus carotinifaciens*. Some aquatic and terrestrial creatures regarded as a primary and secondary sources of the astaxanthin producing and accumulating it through their metabolic pathways. Astaxanthin is the powerful antioxidant, nutritional supplement as well as promising therapeutic compound, observed to have activities against different ravaging diseases and disorders. Researchers have reported remarkable bioactivities of astaxanthin against major non-communicable chronic diseases such as cardiovascular diseases, cancer, diabetes, neurodegenerative, and immune disorders. The current review discusses some structural aspects of astaxanthin. It further elaborates its multiple potencies such as antioxidant, anti-inflammatory, anti-proliferative, anti-cancer, anti-obese, anti-diabetic, anti-ageing, anti-TB, anti-viral, anti-COVID 19, neuro-protective, nephro-protective, and fertility-enhancing properties. These potencies make it a more precious entity in the preventions as well as treatments of prevalent systematic diseases and/or disorders. Also, the review is acknowledging and documenting its powerful bioactivities in relation with the pharmaceutical as well as nutraceutical applicability.

**Graphical Abstract:**

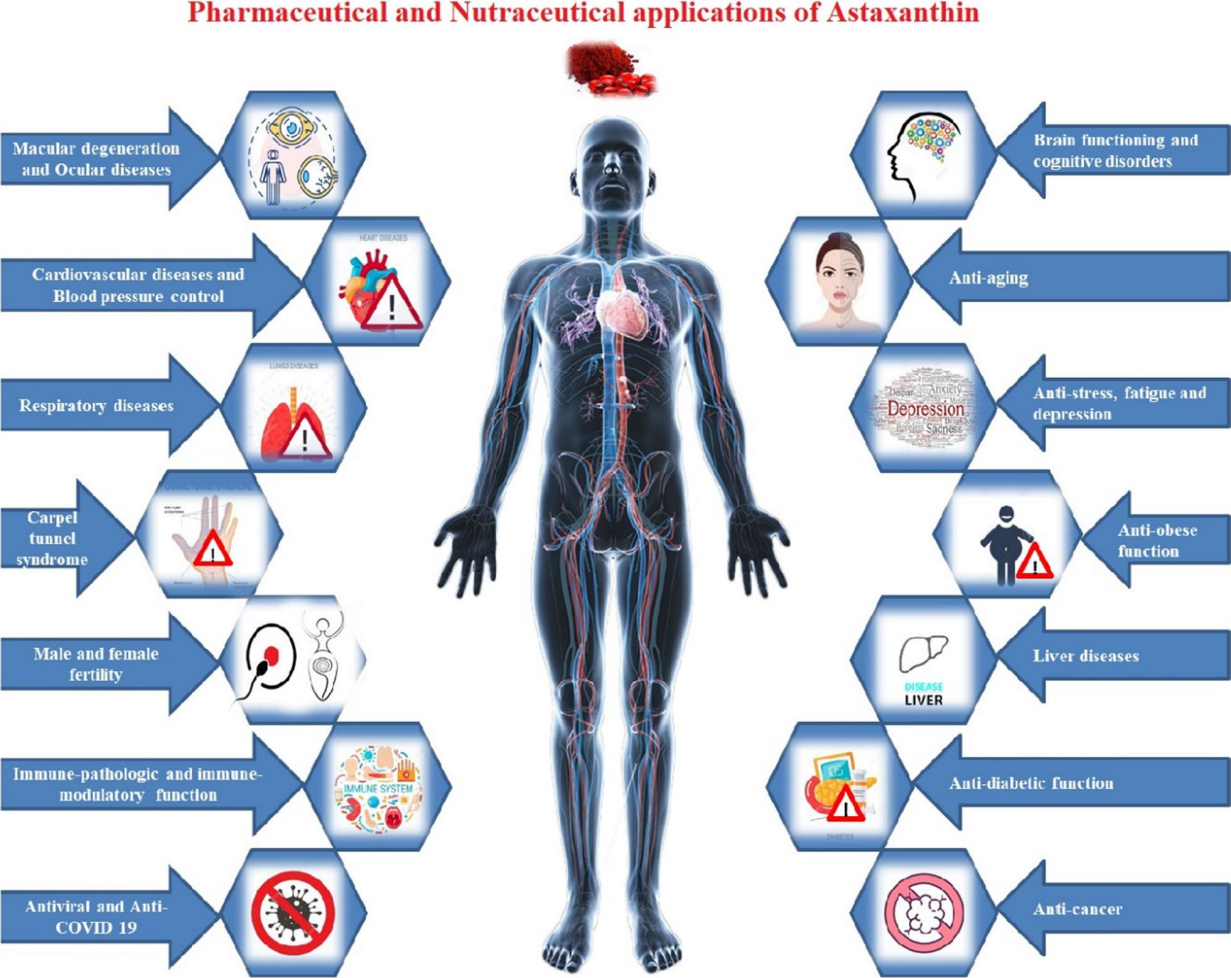

## Introduction

Carotenoids are a large class of bio-pigments found universally in plants, algae, fungi, and bacteria in yellow, orange and red shades. Terpenoids is one of the subfamily of carotenoids containing more than 55,000 varied structures. It is a well-known group of natural secondary metabolites having pharmaceutical, nutraceutical, and seasoning properties [1–3]. Prior studies have shown significant role of carotenoids on human and animal health [4]. Some well-known bio-functions of carotenoids are vitamin A conversion and immunity boost up. It is also useful in the protections by scavenging of free radicals in some lethal non-communicable diseases viz, Cardiovascular diseases, Cancer, Respiratory diseases, Diabetes [5, 6]. Since carotenoids cannot be synthesized by animals and is need to be provided as the dietary supplements for the wellness purposes [7].

In recent times, hectic lifestyle has increased the prevalence of many chronic diseases. Consequently, people are trying to manage their lifestyle habits with the help of some preventive healthcare supplements and that stands a driving force of market growth in pharmaceutical and nutraceutical industries. Emerging economic countries such as China, India and South America have been anticipated for offering growth opportunities, predominantly in the nutraceutical and cosmetic industries for the next six years in the global market [8].

Earlier, people used to have medicines and pharmaceutical products whenever they had some illnesses. However, during the last few years, people throughout the world are becoming more self-aware and health conscious. Such habits of the people have been enhanced more after COVID-19 pandemic. They are now very much curious and are giving more importance to maintaining their good health and wellnesses. With this perspective, the health and wellness industries are going to get uplifted in coming years. The wellness is described as a process that an individual follows to achieve top mental health and physical wellbeing. Which can be attained by developing a good eating habit, along with an active lifestyle. Exactly here the role of certain dietary supplementation get involved. The researchers are actively exploring more and more superfoods in this context [8].

It is being brainstormed, that certain natural bioactive compounds like carotenoids may possesses the potency of fulfilling such nutritional gaps and hence, are getting explored curiously for it. The carotenoid named, astaxanthin (AST) is top listed in those bioactive compounds. Which is a tetra-terpenes with cyclic C_40_ scaffold with eight isoprene units. In global market, AST acquires third position as the most important carotenoid after β-carotene and lutein. Perhaps the trade market of AST has been expected to be valued at USD 647 million in 2021 and estimated to the peak up to USD 965 million by the year 2026 [4].

### Chemistry of astaxanthin

**Fig. 1:**
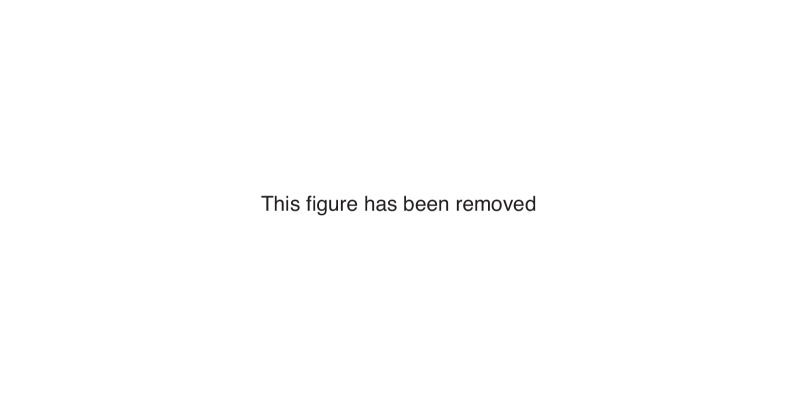
Orientation of astaxanthin, β-carotene, vitamin C and vitamin E in plasma membrane

Astaxanthin is a strong lipophilic keto-carotenoid [3,3′-dihydroxy-β,β′-carotene-4,4′-dione] insoluble in aqueous solutions while soluble in nonpolar solvents such as dichloromethane (~ 30 g/L), chloroform (~ 10 g/L), dimethylsulfoxide (~ 0.5 g/L) and acetone (~ 0.2 g/L) (Table 1). The AST shows close structural resemblance to other carotenoids such as β-carotene, zeaxanthin and lutein. Its absorption spectrum reveals a conjugated polyene structure with λ_max_ of 489, 478, and 480 nm in chloroform, ethanol, and acetone respectively [7]. It is an optically active compound; as its structure is made up of long nonpolar conjugated polyene backbone and two polar β-ionone rings at each side [9]. Each ring holds one hydroxyl (–OH) and one keto (C=O) moiety actually imparts a good antioxidant property to it. The polar-nonpolar-polar linear structural array of the astaxanthin enable it to bind and span the cell membrane. Thus, AST’s chemical structure and its orientation in plasma membrane (Fig. 1) favor counterbalancing of reactive oxygen (ROS) as well as reactive nitrogen species (RONS) [10]. AST is well recognized for its antioxidant potencies. It is 110 times potent antioxidant than vitamin E, 560 times than green tea catechins, 800 times than Co-Q 10, 3000 times than resveratrol and 6000 times than vitamin C [11–13]. It is strongly supposed as a completely unique antioxidant and a potent bioactive compound as it shows three concurrent novel peculiarities i.e., powerful anti-oxidation property, safe for human use and acquires best position within the cell membrane [13].

**Table 1:** Chemical properties of astaxanthin

CAS-No	472-61-7
E-No	E161j
Doc. No. (21 CFR)	73.35
MF	C_40_H_52_O_4_
MW	596.8 g mol.^−1^
Density	1.07 g/cm^3^
MP	216 °C
BP	774 °C

**Fig. 2:**
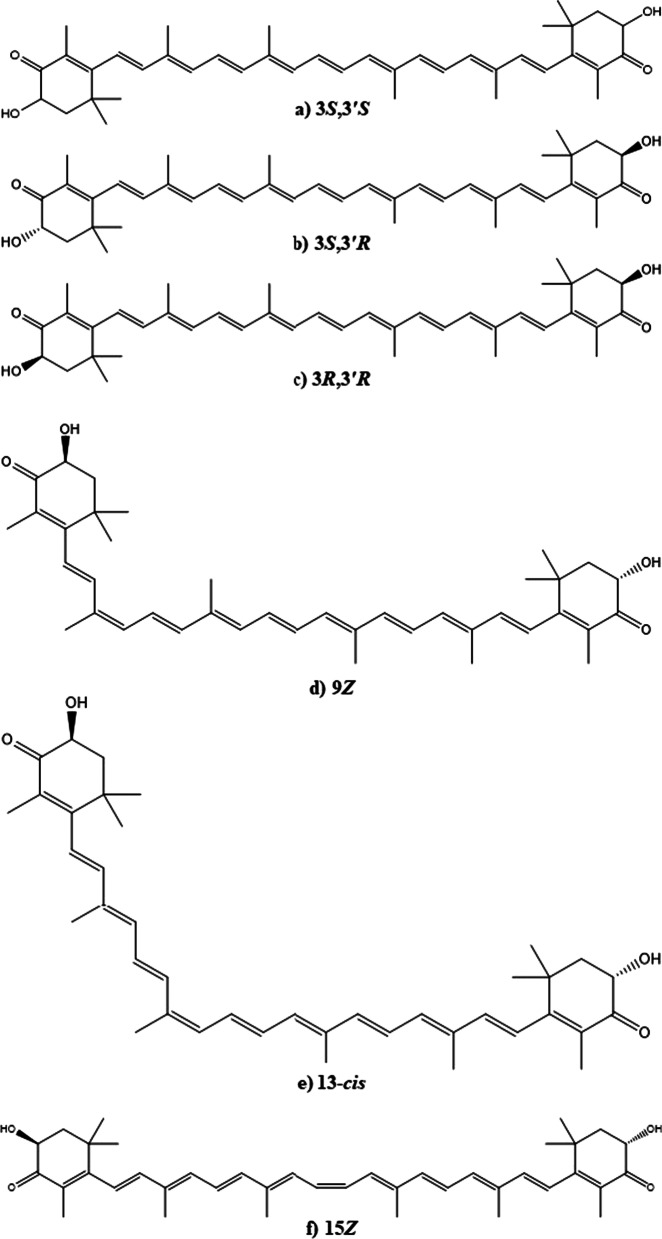
Structures of optical isomers (**a**–**c**) and geometric isomers (**d**–**f**) of the astaxanthin

Structurally, AST is present in *trans* and *cis* (*E* and *Z*) isomeric forms (Fig. 2). Whereas, all *trans* (all *E*-AST) isomers are more stable and therefore those are abundantly found in the nature. Three different stereoisomers namely 3*S*,3′*S*; 3*S*,3′*R* (*meso*); 3*R*,3′*R*; results due to presence of chiral centers at C-3 and C-3′ on β-ionone rings. Amongst them, the all *trans* 3S,3′*S* isomer is commonly seen in nature. Its esterification can be possible because of hydroxyl (-OH) groups on C-3 and C-3′ positions, which enables its increased solubility, absorbability and transport within the cells. Either one or both hydroxyl (-OH) groups can bind to various fatty acids and produce mono or di-esters. AST can also be present in a free form where hydroxyl (-OH) groups remain to be esterified and even it forms a bonding with proteins or lipoproteins in the cell [9, 14]. The change in molecular environment leads to change in an absorbance of AST. For example, in the case of lobsters carotenoprotein complex is observed to be greenish blue rather than reddish orange [9].

### Synthetic astaxanthin

Chemically derived AST is heading the market over natural AST [15]. Basically, the manufacturing of synthetic AST is more complex when it comes to pharmaceutical standards. However, researchers have succeeded in the artificial synthesis of 3*S*,3′*S* enantiomer; mimicking natural AST [16]. Further, derivatives are being developed for clinical trials studies. Disodium disuccinate astaxanthin (DDA), a synthetic form of AST manufactured by Cardax Pharmaceuticals and reported to be used in experimental models of myocardial ischemia and reperfusion [17]. It’s easy to use by intravenous or oral way, because of good aqueous solubility [18]. Since DDA is not available from the manufacturers, another derivative of AST has been launched by Cardax Pharmaceuticals i.e. pro-drug Heptax/XanCor, CDX-085 [19]. The pro-drug was claimed for the treatment of liver inflammatory diseases, reduction of triglycerides, prevention of re-thrombosis and amelioration of the metabolic syndrome [20]. The CDX-085 compound shows greater water solubility as well as bio-availability than DDA and the natural AST derived from *H. pluvialis* [18, 21].

**Fig. 3:**
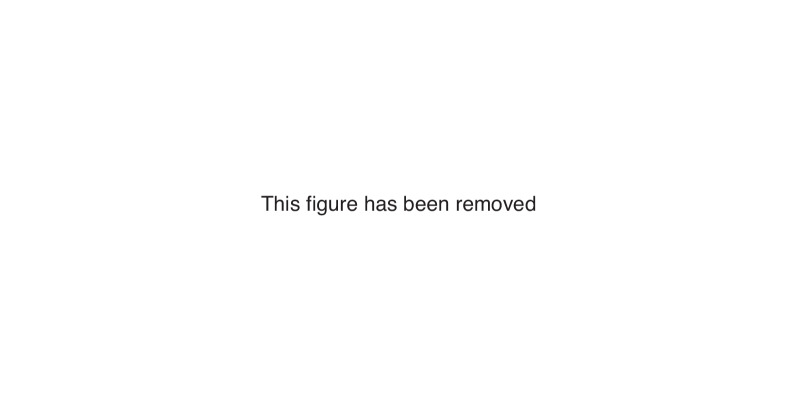
Astaxanthin applications in various industries

Although the synthetic AST has better applicability, people prefer to use natural AST produced by microorganisms using biotechnological processes [22]. Due to the more demand of natural AST by the health conscious people, the industries like pharmaceutical, nutraceutical, feed, and cosmetics use only natural AST because of its biosafety [21] (Fig. 3).

### Natural astaxanthin

**Fig. 4:**
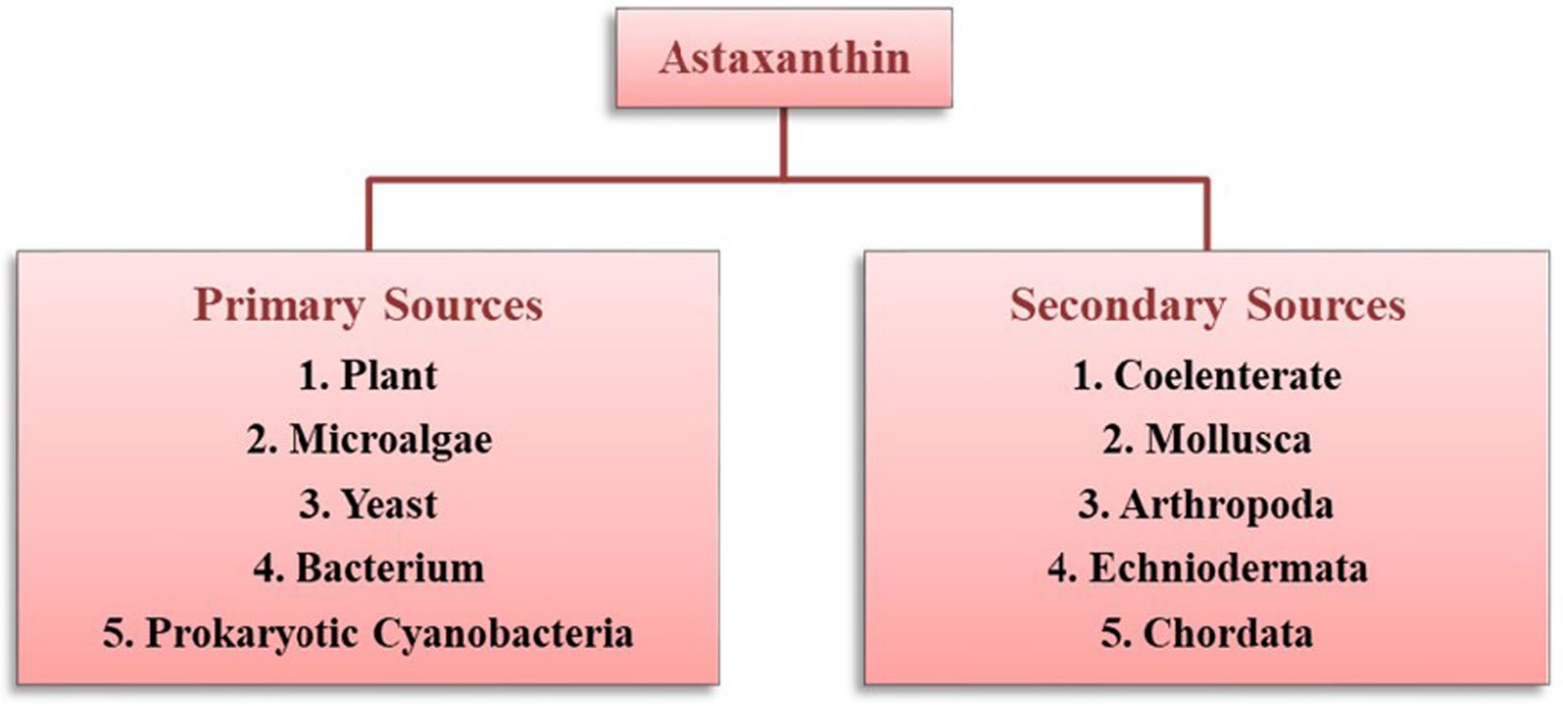
Natural sources of astaxanthin production

The known primary sources of natural AST includes certain, plants, algae, fungi, yeast and bacteria. More precisely, the sources may include flowering plant (*Adonis*), microalgae (*Haematococcus pluvialis*, *Chlorella zofingiensis*, *Chlorococcum* sp.), yeast (*Phaffia rhodozyma*) and bacteria (*Agrobacterium aurantiacum, Paracoccus carotinifaciens*) [14, 23] (Fig. 4). The AST biosynthesis occurs by *de nono* pathway in their natural systems including. AST is ubiquitously found in marine ecosystems like salmon, shrimp, crayfish, lobster, krill, Arctic char, brook trout (red trout), *Pagrus major* (red bream), red snapper (*Lutjanus campechanus*), copepod, and asteroidean. The feathers of birds like quail, flamingo and scarlet ibis could also own AST [21, 24]. These marine animals are unable to synthesize AST by biochemical ways, and therefore they do take and accrue AST from zooplanktons including algae and other microbes [9, 14, 25].

#### Primary sources

The genes required for astaxanthin biosynthesis can be observed in some natural producers such as plant, microalgae, yeast, bacteria, etc. Therefore, these are considered as the primary sources and have been elaborated in the following section.

##### Plant derived astaxanthin

As per earlier reports, AST accumulation was observed in plants of *Adonis* species with an AST biosynthesis pathway wholly distinct from that of bacteria and green algae [26]. The red–orange flowers of the genus *Adonis* from *Ranunculaceae* family are well-known for AST production. The species like *A. aertivalis*, *A. amurensis*, *A. annua* have been studied so far. According to Mawson R., a strain *A. aertivalis* is an abundant source of valuable AST. It can give 200–350 μg of AST from an average of 18–22 petals of a single flower [27]. In *A. annua* flower petals, the amount of AST reported is about 1% of its dry weight [28, 29]. As per the recent studies of Li Y., AST extracted from the *A. amurensis* flower petals was accounted for 3.31% of the dry weight [30].

In addition, one of the classes of Bryophyta known as liverworts (small green terrestrial plants) has been recently reported to produce AST. The AST has been detected in the liverwort gametophyte by a Thin Layer Chromatography (TLC) of the same [31–33]. This may suggest that there might be many more plant sources yet to be explored for AST productions.

##### Microbial derived astaxanthin

*Microalgae, Haematococcus* Among different microbial sources, *Haematococcus pluvialis* is considered as the most promising one; therefore, it is widely exploited for the natural AST production. *H. pluvialis* (class -Chlorophyceae; order- Volvocales; family- *Haematococcaceae*) is a unicellular eukaryotic photosynthetic green microalgae generally found in freshwater [34]. Currently, the highest commercial AST production takes place from the *H. pluvialis* with 3.8%–5% of its dry weight [35–38]. The produced AST is present with 73% *trans*-AST and 27% as *cis*-AST and predominantly in *3S,3′S* form [39–42]. The United States Food and Drug Administration (USFDA) have recommended *H. pluvialis* derived AST as a “GRAS” (Generally recognized as safe) in 1999 and can be considered as a nutraceutical compound for consumptions [43]. However, the European Food Safety Authority (EFSA) has approved daily intake AST for humans is 0.034 mg/kg and can be used in dietary supplements in the form of nutraceuticals [44–46].

*Red yeast Phaffia (Xanthophyllomyces dendrorhous)* *Phaffia rhodozyma* (class- Tremellomycetes; order- Cystofilobasidiales; family- Cystofilobasidiaceae) has also been recognized as another good source of natural AST producers [47]. *P. rhodozyma* is reported to produce 0.5% AST of its dry weight [48, 49]. Generally, free and non-esterified 3*R*,3′*R* AST is synthesized. The red yeast’s AST is considered as GRAS by USFDA and has been certified as a colour additive in fish feed supplements by FDA [38, 50, 51]. Recently, another yeast (*Rhodosporidium toruloides*) was reported as a new source of AST and in coming years, the source will be more explored for its productions and other properties [46, 52].

*Bacteria:*

*Paracoccus:* *Paracoccus carotinifaciens* (class- Alphaproteobacteria; order- Rhodobacterales; family- Rhodobacteraceae) is an anaerobic Gram-negative bacterium that shows a wide range of carotenoid synthesis abilities [53]. *P. carotinifaciens* is reported to produce 2.2% AST of its dry weight in non-esterified form [54, 55]. The stereo-isometric form i.e., 3*S*,3′*S* produced by this bacterium is similar to that of *H. pluvialis* form. And many human trials revealed that bacterium derived AST is considered as safe for human consumption [45, 46, 56]. So the source is being more explored by multiple research groups for enhanced productions and more applicability.

*Prokaryotic cyanobacteria: *AST was also found in a limited number of cyanobacteria. It is synthesized from β-carotene via zeaxanthin and canthaxanthin by carotene β-ketolase (*BKT/CrtW/CrtO*) and *CHYB/CrtZ/CrtR* [57].

#### Secondary sources

Secondary sources of AST includes some aquatic and terrestrial animals, which cannot synthesize it through their metabolism. However, they can acquire it from their food chain. Some of the secondary sources of AST have been highlighted in the following section.

##### Coelenterate

AST is observed in tissues of certain marine animal, which actually originates from their dietary zooplankton [58, 59]. Two unique metabolites of AST i.e., 2-nor-astaxanthin and actinioerythrin were reported in sea anemones, *Actinia equina* and *Tealia feline* [60].

##### Mollusca

In a freshwater snail *Pomacea canaliculata*, an experiment with feeding of carotenoids have showed that the β-carotene was oxidatively metabolized to AST (3S,3ʹS) [60, 61]. In the food chain of algae to sea angel, *Clione limacina*, AST is found to be produced from the oxidative metabolism of β-carotene, β-cryptoxanthin and zeaxanthin. However, 7,8-didehydroastaxanthin and 7,8,7ʹ,8ʹ-didehydroastaxanthin were seem to be synthesized from diatoxanthin and alloxanthin respectively [60].

##### Arthropoda

Many crustaceans can also synthesize AST from β-carotene, ingested in dietary algae, via different metabolic intermediates such as echinenone, 3-hydroxyechinenone, canthaxanthin, and adonirubin. In *Penaeus japonicas*, racemization of AST was observed when these prawns were fed with [^3^H]-labeled AST (3*S*,3ʹ*S*) [62]. As a result, the isotopic AST converted to (3*R*,3ʹ*R*)-meso, and (3*S*,3ʹ*S*)-isomers at an approximate ratio of 1:2:1. Among this metabolic conversion, iso-astaxanthin (4,4ʹ-dihydroxy-ε,ε-carotene-3,3ʹ-dione) was the key intermediate [60].

In Orthoptera, particularly grasshoppers do possess AST and adonirubin as mixtures of optical isomers [63]. However, in locusts, it was synthesized from β-carotene.

In Lepidoptera, in the pupae of swallowtail *Papilio xuthus*, zeaxanthin metabolism results in AST (3*S*,3ʹ*S*) via adonixanthin. Whereas orange green shade of pupae is possibly due to AST, fritschiellaxanthin, and papirioerythrinon metabolites [64].

In spider *Nephila clavata* (Arachnida), AST is present as one of the major keto-carotenoid [65]. As per the report of Veeman, two-spotted spider mite i.e. *Tetranychus urticae* contains a series of keto-carotenoids such as adonirubin, astaxanthin and 3-hydroxyechinenone [66]. During long nights of lower temperatures, when female spider mites enter into a facultative diapause condition characterized by the termination of reproduction, visible changes in the body colour from faint yellow to red–orange shade occurs. Such transition results due to the accumulation of keto-carotenoids, such as AST. Hence, AST was proposed to protect from physical stress caused by overwintering [60].

As per the recent reports of Wybouw and co-workers, the knockout technique revealed that the endogenous CYP384A1 gene of *Tetranychus urticae* (plant feeding mite) is positively code for carotenoid ketolase, which synthesizes AST [33, 67].

##### Echinodermata

Starfishes are carnivorous in nature and they feed on small crustaceans and bivalves. In the marine system, carotenoids mainly transfer from zooxanthellae (dinoflagellate algae) to starfish via corals. The starfish *Acanthaster planci* eats corals. Whereas, coral *Acropora japonica* shows symbiotic association with zooxanthellae and so carotenoids easily get absorbed from them and accumulate in corals without any changes. By the end of these oxidative metabolic transformations, 7,8-didehydroastaxanthin and AST were found as major keto-carotenoids in starfish [59, 68, 69].

AST was also observed in the gonads of sea cucumbers. One of the known sea cucumber *Plesiocolochirus minutus* was found to own 9Z,9ʹZ configured tetrahydroastaxanthin and which has been considered to be a metabolite of AST [70].

##### Chordata

Oxidative metabolism of zeaxanthin in Cyprinidae fish leads to the production of AST (3*S*,3ʹ*S*) via adonixanthin and idoxanthin [60]. Whereas in Reptilia, it was found in various species of lizards. While in the zebra finch, it was a major carotenoid in feathers and beak along with other keto-carotenoids, such as 4-ketolutein, adonirubin, and canthaxanthin [71]. The red colour shade in the feathers of American flamingos is due to AST, derived from dietary micro-crustaceans [72]. Among different carotenoids, it was also observed in the red plumage of caciques and meadowlarks (Icteridae) [71].

The pompadour cotinga (*Xipholena punicea)*, a species from the Amazonian rainforest shows interesting structural carotenoids such as 3-methoxy-4-keto-β-ring and 2,3-didehydro-3-methoxy-4-keto-β-ring moieties along with canthaxanthin and AST [73, 74].

## Astaxanthin biosynthesis factors

Primarily β-carotene forms a precursor for AST synthesis and is catalyzed by β-carotene ketolase and hydroxylase to canthaxanthin and zeaxanthin metabolites respectively. Previous investigations say that, declined photosynthetic activity or a limited oxygen evolution triggers accumulation of AST. The damaged PS-II complex generates limited O_2_ which acts as the principal factor for AST pigment synthesis [75–79]. Therefore, the amount of AST production is inversely proportional to photosynthetic events, despite the amount of chlorophyll and PS-II reaction center remains constant [78, 80].

The photosynthetic inequity between input energy from the light adsorption by an antennae and output energy in the form of CO_2_ fixation gets quenched and it produces reactive oxygen species (ROS). So, earlier studies reveals that the carotenoids certainly will prevent excessive injuries caused by ROS; directly quenching triplet chlorophyll (3-Chl) or singlet oxygen (^1^O_2_) produced from photodynamic reactions [81–83]. In contrast, when CO_2_ fixation gets restricted by stress, environmental surroundings such as nutrient famine, cold/high temperatures, high salinity or low CO_2_ accessibility, the production of these ROS could occur even at moderate light intensity because of an energy spare [84, 85]. While under nutrient starvation, O_2_ is possibly the most effective ROS species that might take part for AST accumulation [86, 87]. ROS could also stimulate the expression of genes coding for carotenogenesis enzymes [88, 89].

The higher light intensity and nutrient stress; permutated with NaCl or sodium acetate had shown boosted production of total carotenoids and total AST content to 32 mg/g and 24.5 mg/g of dry biomass, respectively [90, 91].

## Pharmaceutical potential of astaxanthin in chronic diseases

The strong antioxidant property of AST has been allied to various biological functions was shown in animal and human clinical trials. It has promising applications in human health and nutritions [92–96]. Some of the remarkable applications of it have been discussed below.

### Cardiovascular diseases

Cardiovascular diseases (CVDs) are a prominent cause of deaths worldwide. According to WHO, about 17.9 million people died from CVDs, signifying 32% of all global deaths, in 2019. Among those, 85% of deaths were simply due to heart attack and stroke [97].

Many risk factors eventually contribute to CVDs such as obesity, hypertension, dyslipidemia, glucose metabolism dysfunctions, oxidative stress, elevated levels of reactive oxygen species (ROS) and reactive nitrogen species (RONS) and inflammation [98–100]. But these risk factors can be controlled by managing and adapting healthy lifestyles, correcting biological clocks and having supplements enriched nutritious diets. As per earlier reports, the oxidative stress and inflammations are correlated and mutually contributed in the initial dealings of the CVDs. Those can be restricted by the influence of antioxidants like AST, which can modulate redox balance, regulate inflammatory responses and control lipid as well as glucose metabolism [101–105]. Such significant abilities of AST could help in preventing disorders like dyslipidemia, arterial hypertension and atherosclerosis as well [106].

Molecular and cellular mechanism of actions of AST has revealed its potential in preclinical and clinical studies in prevention and treatment of CVDs. By reducing mitochondrial and cellular oxidative stress, elevating antioxidant cascades and related enzymes, protecting blood rheological parameters, equilibrating mitochondrial dynamics, biogenesis and mitophagy, it can manage homeostasis too [10, 107, 108]. It also possesses the potential worth in setting of ischemia–reperfusion and controls glucose as well as lipid metabolism by reducing lipid peroxidation and re-thrombosis after thrombolysis [105, 109, 110].

### Blood pressure control

Administration of AST has lowered BP in the rats, which was elevated there due to sucrose intake and insulin resistance [111]. Consequently, in hypertensive rats (SHRs) observed for lowering of blood pressure and hence delay in the frequencies of strokes [112, 113]. However, this antihypertensive effect is still not fully known and it needs to be studied comprehensively in future [105].

According to recent report of Mashhadi N.S., AST supplemented in type 2 diabetic patients were recorded for increased glucose metabolism, lowered visceral fat deposition (11.2 ± 3.4 vs. 11.85 ± 3.8% compared with placebo; P < 0.05) and decreased systolic blood pressure (132 ± 18 vs. 133 ± 19 and 143 ± 27 mmHg compared with placebo and baseline, respectively; P < 0.05) [114]. The factors such as blood plasma velocity and vasodilation can directly affect the vascular resistance, possibly contributing to hypertension and producing cardiac complications such as myocardial hypertrophy [115].

In experimental studies on SHR rats shown reduced systolic blood pressure and persuaded significant histological alterations in the aorta along with decreased vascular stiffness when supplemented with AST [102, 108, 116]. However, in stroke-prone SHR rats, oxidative stress marker like 8‑hydroxy‑2'‑deoxyguanosine (8‑OHdG) effectively down regulated. Henceforth, dropped systolic blood pressure was observed with repressed thrombogenesis in the cerebral veins [117].

However, synthetic AST i.e., CDX-085 pro-drug administration in murine model of thrombosis has shown increased blood flow of the carotid artery up to ~ 20% prior to manifestation of endothelial dysfunction [10, 19].

### Anticancer activity

In current scenario, cancer is most perilous cause of death across the globe. It is accounted as second deadliest disease [118]. According to GLOBOCAN reports, an estimated incidences and mortality are constantly growing every year [119]. From many decades, different therapies and strategies are in use to combat against cancer. Among them, natural products based pharmaceuticals are gaining accomplishment in cancer treatments. As per FDA norms, almost 547 natural bioactives and their derivatives have been sanctioned for preclinical, clinical phases I to III, and preregistration studies. They basically originate from plants (47%), bacteria (30%) and fungi (23%) [120, 121].

Oxidative stress and related cellular damages such as DNA mutations and protein anomalies are the root causes of carcinogenesis. Along with other natural products, research scientists have revealed the anticancer role of different carotenoids. Among them, AST was discovered as a strong bioactive showing contribution in prevention and/or treatment of malignant cells [121]. In early 90 s’ its anticancer potential was seen against well-known genotoxic hepatocarcinogen i.e. aflatoxin B1, by studying detoxification mechanisms [122]. Where it showed maximum free radical scavenging activity [123], superoxide reductions and nitric oxide generation properties [124]. In another studies, AST suppressed the DNA damage when exposed to UV-A via its antioxidant cascades [125]. A successive 5 days treatment with 50 μM of AST to the mouse epidermal cell line JB6 P +, it displayed significant anti-neoplastic effects and decreased viability up to 94% [126]. Later, Chen et al. examined anti-proliferative activities of AST (168 μM) on two melanoma cell lines. In both cell lines proliferation inhibitions were observed, up to 50% in A375 and 80% in A2058 cell lines [127]. Similar studies were performed further by another group on rat breast cancer cells (SHZ-88), mouse lewis lung carcinoma cells and human hepatocarcinoma (CBRH-7919). In all the three cell lines, CBRH-7919 was shown higher IC50 value (39 μM) [121, 128]. In addition, AST has induced cell cycle arrest in mice H22 hepatoma cells in-vitro and in-vivo both and showed minor impact for apoptosis induction [129]. Besides, combinatorial effect of AST with β-carotene (shrimp) and lutein (plants) synergistically induced apoptosis by modulating expressions of cyclin D1, BAX, p53 and Bcl-2 in MCF-7 breast cancer cells [130, 131].

Ko et al. have checked AST’s anti-proliferation effect on two human non-small cell lung cancer (NSCLC) cells viz., bronchioloalveolar cell carcinoma (A549) and squamous cell carcinoma (H1703). AST with minimal concentration (20 μM) had shown a reduction in cell proliferation with 50.9% and 39.71% in A549 and H1703 cells respectively [132]. On the other hand 42 μM of the same has inhibited growth of the liver cancer cell line (HepG2) [133]. While in colorectal cancer cells (LS-180), AST induced apoptosis cascade mechanism by triggering BAX and caspase-3 expressions and inhibited growth and proliferation by suppressing Bcl-2 expressions. The activity was correlated to the strong antioxidant activity of AST imparting increase in the levels of superoxide dismutase, catalase, glutathione peroxidase and decreased levels of malondialdehyde [131, 134].

Recently, another review report was produced by a group emphacising an anticancer effects of AST by multiple molecular mechanisms including the role of AST in certain *in-vitro* anticancer studies, cell cycle arrest in tumor cells, various pro-apoptotic and chemo-sensitizing effects, suppression of cancer diffusion and pre-clinical studies [121]. The earlier literature can easily highlight and qualify the AST as one of the best natural drug combating with cancer.

### Respiratory diseases

Respiratory diseases arise due to impaired or disturbed lung functioning. Regular respiratory activities such as breathing and pulmonary functions are majorly get affected. Some of the well-understood causes of lung diseases are bacterial, viral or fungal infections. Other lung diseases are allied to environmental factors and which possibly leads to asthma, acute lung injury (ALI), chronic obstructive pulmonary disease (COPD), emphysema, lung fibrosis, mesothelioma, lung cancer and non-small cell lung cancer (NSCLC) [135].

AST has been found to be a potent detoxifier and oxidative stress reliever in smoker community [136]. Smoking induces more free radicals which lead to an increase in oxidative stress levels and make a person prone to certain secondary health problems such as respiratory diseases, cardiovascular diseases, strokes and many more. Earlier studies have advised that the active smokers do need a higher daily intake of antioxidants than the passive ones to diminish the consequences of extensive and continued exposure to cigarette toxins by them [10].

A successive 3-week administration of AST (5 mg, 20 mg and 40 mg) in persistent smokers was observed to decrease the oxidative damage by suppressing the lipid peroxidation process. The parameters like declined malondialdehyde (MDA) and isoprostane (ISP) levels in blood serum, elevated superoxide dismutase (SOD) and catalase (CAT) activity and overall antioxidant activity have confirmed the antioxidation mechanism induced due to AST administration upto certain threshold concentrations [136]. Beyond the same concentrations, the results were not altered meaningfully. This may suggest that at higher dose the AST might have saturated [10].

Recently, Cheng J. and Eroglu A. have reported the effects of AST on lung diseases such as asthma, acute lung injury (ALI), chronic obstructive pulmonary disease (COPD), emphysema, lung fibrosis, lung cancer and non-small cell lung cancer (NSCLC). Their study has also summarized the molecular mechanisms of those effects. Therefore, the AST has provided an insight on future prospective and direction of using as a potent bioactive nutraceutical in clinical trials with respect to the lung diseases [137].

### Anti-diabetic function

Diabetes mellitus (DM) is a chronic hyperglycemic (HG) disorder resulting from an impaired carbohydrate metabolism like insufficient insulin secretion, its action, or both. The DM has three main categories, type 1 (T1DM), type 2 (T2DM) and gestational DM. Among these, T2DM has accounted for about 90% of all the diabetes across the globe. In T2DM, an uncontrolled and long term increased blood glucose level develops life-threatening health issues such as cardiovascular diseases, retinopathy, nephropathy, neuropathy, gangrene and other related complications [138]. The root causes of DM and its complications are linked to oxidative stress and chronic inflammations [139] along with the hampering of cellular homeostasis and its subsequent complications such as vascular dysfunction, etc. [140, 141].

Scientists have reported that there could be a major role of nutritional and pharmacological therapies in the clinical management of diabetes. Wherein, various antioxidants including carotenoids, have been intensively screened for their effects in the prevention of progression in diabetes and its complications [114, 142]. In particular, it has been reported that the AST could significantly reduce the postprandial hyperglycemia in alloxan-induced diabetic mice [143]. Similar observations were pointed out in the pancreatic β-cells, in which oxidative stress induced by hyperglycemic conditions [144, 145].

In another study, a supplementation of the AST (8 mg/day for 8 weeks) in mice has shown that the adiponectin (an adipose-specific protein) can play a protective role in insulin resistance/diabetes. Subsequently it could also reduce the fructosamine and hypo-adiponectinemic conditions, causing increased plasma glucose regulation and fatty acid oxidations [146, 147]. Whereas in T2DM diabetic db/db mice models, AST has protected β-cells of the pancreas from glucose toxicity by reducing oxidative stress and regulating blood glucose levels. In other animal studies, AST demonstrated promising activities in reducing hyperglycemic concentrations via optimizing insulin secretions, regulating glucose metabolism and GLUT4 functions [148, 149].

In type 2 diabetic patients, AST relatively reduced TG, VLDL cholesterol levels, blood pressure, insulin resistance and also shown vasodilation properties [106, 116, 150]. Furthermore, AST demonstrated a slight rise in HDL levels and which was positively correlated to changes in adiponectin concentrations. But still, the mechanism remains unclear [113]. A similar study revealed a likely association between serum adiponectin and HDL cholesterol concentration [151]. Yoshida et al. reported, 12 mg AST supplementation in non-obsess volunteers significantly increased HDL cholesterol concentration and was in with positive correlation to adiponectin levels [106, 114].

The animal model studies with an uptake of AST (35 mg/kg) for almost 12 weeks displayed a correlation between dropped glycemic index and oxidative stress biomarker i.e. malondialdehyde (MDA) content with increased SOD activity in the blood serum [152]. And when AST (1 mg/day) was administered for 18 weeks different observations were recorded which explain the reduction in non-fasting glucose levels with substantial protective action against glucose toxicity in the pancreatic cells which were capable of producing insulin [144]. In another study, AST prohibited impaired functioning of pancreatic β-cell by defending them from the endoplasmic reticulum (ER) stress-induced apoptosis via regulating expressions of c-Jun N-terminal kinases (JNK), PI3K/Akt pathways and ER chaperons [152].

The studies performed in HFFD (high-fat fructose diet) fed mice; daily supplementation of AST (6 mg/kg per day) for about 45 days revealed increased insulin sensitivity and decreased plasma glucose as well as insulin levels [153]. An analogous report in HFFD mice showed augmented glucose metabolism by increased assimilation of glucose in body muscles [154]. Whereas in streptozotocin-induced diabetic rats, AST (0.01% or 0.05%) administration via diet reported declined proportion of activated macrophages (MCP-1) and inflammatory factors such as IL-6 and TNF-α in the plasma [141, 155].

An inclusive data from reviewed literature clearly depicts the potency of AST in the management of diabetes. Prior studies on T1DM and T2DM revealed several mechanisms of action of the same. Hence, AST can be regarded as a safe nutraceutical compound for an effective prevention of DM and DM related complications [141].

### Anti-obese function

In the current scenario, obesity is also a globally concerned health issue. It is threatening people to become prospected sufferer from the health hazards such as hyperlipidemia, cardiovascular diseases, hypertension, and type II diabetes. AST supplementation via food has reported less weight gain, reduction of plasma cholesterol and liver triacylglycerol (TAG) in animal models [156]. In other studies, it was also considered to boost up the lipid metabolism during exercise of an individual leading to reduction in the body fat and increment in muscular movements [157]. However, in the actual human studies performed by few researchers, it did not show promising results in reducing body fat or body mass index [158].

Chio et al. demonstrated antioxidant effects of AST in obese individuals. The subjects who had received 5 and 20 mg of AST for successive 3 weeks showed subordinate levels of oxidative stress biomarkers which are linked to lipid peroxidation. When compared with the reduction in malondialdehyde (MDA) and isoprostane (ISP) levels, increased superoxide dismutase (SOD) and total antioxidant activity was observed [159]. Later 12 weeks of supplementation with 20 mg AST was also found promising in reducing the risks of heart attacks. That is due to an overall decrease in lipid profile including LDL concentrations, ApoB and ApoA1/ApoB [160].

In total, multiple studies have concluded that the AST could be helpful in reducing oxidative stresses, in curbing lipid profiles among obese individuals and ultimately in diminishing the emergence of CVDs in corresponding sufferers [10].

### Liver diseases

The non-alcoholic fatty liver disease (NAFLD) is one of the key contributors to chronic liver diseases, worldwide. About 10% to 20% of them probably evolve into non-alcoholic steatohepatitis (NASH). Gao et al. have revealed protective effects of AST in NAFLD with different mechanisms of actions such as improvement in mitochondrial oxidative respiration, reduction in oxidative stresses, inflammation, liver fibrosis and liver tumor formation. It has also been proven helpful in enhancement of insulin metabolism, stimulation of M2 macrophage polarization and regulation of lipid metabolism [161].

Wu L. and co-workers have investigated an attenuation effect of AST on hepatocyte damages and mitochondrial functional defects generated due to NAFLD. *In-vivo* studies on mice showed upregulation of the FGF21/PGC-1α pathway in damaged hepatocytes suggesting a protecting role of AST in NAFLD treatment [162].

In alcoholic liver diseases (ALD), the influence of AST has been checked in binge alcohol feeding animal models by the researchers. The hepatic steatosis and inflammation, as well as certain consequences rising due to heavy ethanol (EtOH) consumption could be appeased by AST intake. Also, the levels of aspartate transaminase and alanine transaminase levels could be dropped. The ethanol triggered expression of cytochrome P-450 2E1 (CYP2E1), pro-inflammatory factors, cytokines, chemokines and ROS levels could also be lowered. Moreover, decreased infiltration of neutrophils was also observed there. In detailed studies, the AST has revealed its protective actions in ethanol-stimulated hepatic damages by blocking the STAT3 pathway and thus constraining oxidative stress and inflammatory reactions [163].

Liu et al. have attempted to reveal the relationship between gut microbiota of AST fed mice and AFLD improvement. In that studies the AST and ethanol-fed C57BL/6J mice were assessed for their AFLD status and gut microbiome composition was assessed. The results were found to be significant as they inflammation was relieved and unnecessary lipid accumulation was decreased. It also lowered the concentration of serum markers of liver injury. In gut microbiome analysis, it has decreased species from the phylum *Bacteroidetes* and *Proteobacteria* and also the species from genera *Butyricimonas*, *Bilophila* and *Parabacteroides,* the has increased number of species from phylum *Verrucomicrobia* and *Akkermansia* in comparison to EtOH fed group. The study also concluded that the *Akkermansia* could be a potential source for AST induced management studies of AFLD. AST significantly reversed the action of EtOH altered inflammatory and related metabolic parameters. Furthermore, it transformed 18 and 128 Kyoto Encyclopedia of Genes and Genomes (KEGG) pathways involved in lipid metabolism and xenobiotic bio-degradation and metabolism at levels 2 and 3, respectively [164].

In another recent studies, the AST supplementation resulted in the regulation of hepatic cholesterol metabolism via enhanced expressions of certain genes including 3-hydroxy-3- methyl glutaryl CoA reductase, LDL receptor and sterol regulatory element-binding protein 2. Furthermore, two major genes involved in lipid β-oxidation i.e. acyl-CoA oxidase and carnitine palmitoyltransferase-1 were also found to be upregulated, whereas lipogenesis related genes were not significantly affected [165, 166]. In lipid profile meta-analysis (TC, LDL-C, HDL-C and TG) studies, AST showed dropped HDL-C levels but other lipid compositions remained unchanged. Therefore Ursoniu et al. confirmed that AST did not exert any effects on dyslipidemia [158, 167]. Therefore, overall results of many studies have highlighted promising effects of supplementation of AST to the liver disease patients. It could be a new promising area and can avail good hopes in the treatment strategies of liver diseases.

### Autoimmune hepatitis

Li et al. investigated the protective role of AST in immunocompromised Balb/C albino mice. Concanavalin A (ConA) (25 mg per kg) was used as an autoimmune hepatitis inducer. Prior to ConA injection, AST was orally given twice a day for 2 weeks. After detecting levels of serum liver enzymes, all histopathological parameters such as, inflammatory cytokines and other marker proteins, it was revealed that AST helps in the reduction of ConA induced hepatic injury. The possible mechanism was deduced to be down-regulation of JNK/p- JNK-mediated apoptosis and autophagy pathways [168]. So the AST which is having multiple potentials could again passed for a best possible candidate in treatment of autoimmune hepatitis.

### Immunopathologic and immunomodulatory function

Dhinaut and co-authors recently reported an effect of lifetime dietary supplementation of AST on immunochallenged insect model i.e., mealworm beetle (*Tenebrio molitor*). The results have shown a reduced immunopathological damages and improved life endurance by immune depressive effects. The in-vitro and short-term in-vivo studies emphasized an immune-stimulating and antioxidant properties of the AST have shown positive effects. But, the reason behind and to what extent the supplements can perform the functions for long term are still unknown [169].

Some researchers have studied the comparative effect of AST and β-carotene on lab animals. The results showed higher immuno-modulating effects for AST than β-carotene [170]. In the case studies on older animals, when AST was given through a regular diet, it showed enhanced antibodies production with a decreased humoral immune response [170, 171]. AST treated human cells displayed boosted immunoglobulin production at the laboratory level [172].

According to a report, an eight-week of dietary supplementation of AST in humans have [173] showed a significant increase in levels as well as the activity of natural killer cells in blood. Also the DNA damage biomarkers were observed to be decreased. The levels of T and B cells were got amplified and the concentration of C-reactive protein (CRP) was significantly lowered in the AST supplemented group [174–177].

Therefore, it can be concluded that the AST supplementation may enhance the health of a human with immuno-alterations.

### Anti-aging function

Biological as well as photoageing (UV rays exposure) are the two main reasons for age-related changes in the skins of human beings. Particularly in photoageing, UV light exposure causes degradation of extracellular matrix components such as collagen and elastin. It results in ROS production, pigmentation, wrinkle formation and deterioration of skin texture [178, 179]. In such cases, the role of carotenoid intake especially the supplementation of AST (photo-protective pigment) has proven promising for nurturing the skin physiology [45]. Another study summarizes the positive impact of AST in maintaining skin elasticity, reducing wrinkle formation, preserving epidermal barrier integrity and ultimately controlling the skin ageing-related processes [180–184].

### Brain functioning and cognitive disorders

In concern to the brain and nervous system is functioning, an increased influence of oxidative stress and age factors may result in different types of cognitive disorders or diseases. Those can be an Alzheimer’s disease, Parkinson’s disease, Huntington’s disease, Autism and Amyotrophic Lateral Sclerosis (ALS), etc. [185–187].

Mental health diseases come under the class of cognitive disorders. Which deals with the brain and mental functioning such as memory, perception, learning and problem-solving abilities. Major cognitive disorders are dementia, amnesia and delirium. Among them, dementia is known for the gradual decline of brain functioning such as memory impairment, loss of concentration and confusion. Whereas amnesia is a memory disorder in which loss of short term memory hampers the daily routine of the affected person. Delirium is a serious confusion state and is characterized by jumbled thinking and a sedentary state. The cancer patients who have gone through chemotherapy are probably induced to chemobrain, a cognitive impairment. Such patient could lose their quality of life due to memory loss and unable to concentrate properly. These impairments were found to be connected to deterioration of neuronal integrity at the hippocampus region and frontal lobe of the brain [188, 189].

Recent studies showed, powerful protective effects of AST on the human brain as it readily clears the border of the blood–brain barrier (BBB) and mitochondrial membrane. Due to its unique property, the brain and nervous system are considered the chief target organs for AST. Consequently, AST could reduce dysfunctions associated with neurons and Central Nervous System (CNS) [189–191].

In current times, AST has been investigated for its protective role particularly in minimizing cognitive impairments ensued by the doxorubicin drug (DOX). In another study, enhanced memory functions and neuro-protective functions by suppressing oxidative stress and several pro-apoptotic stimuli and even bringing optimum acetylcholinesterase activity was seen [192]. In other body parts, it also showed positive effects and cumulatively works on neuro-protective functions and overall brain health. An intake of keto-carotenoid rich diets, particularly AST had shown lowered phospholipid hydroperoxides (PLOOH) levels causing elevated O_2_ transport to the brain by improving RBCs antioxidant status and diminishing chances of dementia [193–196].

Researchers have also evaluated the combinatorial effect of AST and fish oil on brain health by observing the reduction of harmful effects on polyunsaturated fatty acids (PUFAs) [197–199]. The experimental studies on Wistar rats supplemented with 1 mg/kg of AST and fish oil showed lipid safety deeds at the anterior forebrain. The antioxidant activities (TAEC and FRAP) were appeared to increase in rats fed with AST and fish oil together but unchanged in fish oil alone. In total, AST via dietary supplements could increase antioxidant cascades by reducing oxidative stress and also elevating cognitive behaviors through memory acts and positive mood swings [189, 200, 201].

In the case of cerebral ischemia/reperfusion (IR) and neuropathic pain (NP), multiple in-vivo studies have shown protecting and preventing effects with varied mechanisms of actions elucidating potency of AST as a novel neuropathic drug for treatment [202–207].

Amyotrophic Lateral Sclerosis (ALS) is known as progressive nervous system disease. Particularly affects upper and lower motor neurons in the brain and spinal cord, leading to loss of muscle control. According to prior studies, mutations in the gene encoding Cu/Zu superoxide dismutase 1 (SOD1) is the most common cause of ALS. More than 110 mutations have been studied and described to date, in this regard. The regulatory role of SOD1 is to keep the cellular homeostasis of ROS, but a mutated form of SOD1 lacks its function due to structural instability leading to toxic functional gain in forming ALS [208, 209].

In ALS, increased oxidative stress may collapse cellular functions and ultimately causes cell deaths. The effects of several antioxidants have been checked on SOD1 inhibitor [like diethyldithiocarbamate (DDC)] treated rat spinal neurons. The results have proved that the antioxidants such as L-ascorbic acid, L-histidine, α-tocopherol, β-carotene and AST could inhibited the DDC action and rescued the motor neurons from excessive oxidative stresses and thus surviving the neuronal functions. Among these, AST had shown the most promising effects even at its low concentrations (100 nM) than other antioxidants (1 mM) studied [189, 210].

Inclusively, AST can serve as a potent antioxidant and neuro-protective drug which can combat neuro-degenerative diseases by a different mechanism of actions such as anti-apoptosis lowers ischemia by impelled apoptosis, reduction in cerebral infarction in brain tissue, reduction of glutamate release and reduce free radical damage [185–187].

In this way, the AST has crowned to be one of the most promising prospected drug molecule in treatment of brain functioning and cognitive disorders.

#### Alzheimer’s disease

Alzheimer’s disease (AD) is measured as a most chronic neuro-degenerative disorder particularly known for memory impairment due to neuronal loss in the hippocampus and neo-cortex regions. Scientists have reported the nutraceutical role of AST in the protection as well as prevention of age-related central nervous system diseases and disorders [184, 211]. In-vivo studies on Wistar rats mimicking Alzheimer’s disease model supplemented with AST powder extracted from shrimp shells (*Litopenaeus vannamei*) displayed effective improvement in cognitive functions [212]. In another latest research, the effect of AST on cognitive modulations was compared with that of the composite derivative made up of AST with docosahexaenoic acid (DHA). The DHA-acylated AST ester (AST-DHA) was accessed for the neuroprotective actions in APP/PSEN1 (APP/PS1) double transgenic mice. Which has diminished the cognitive malfunctions [207, 213] and hence, it could be considered a potential therapeutic agent for the Alzheimer’s disease.

Several existing studies have elucidated vigorous effects of AST on human brain health, neurogenesis and plasticity generation, particularly in the elder age groups [214, 215]. In amyloid-beta plaques induced oxidative stress, inflammations at the neurofibrillary web junctions cause neuronal death incentives in the brain of AD suffering patients. In such studies, AST supported strong antioxidant, anti-inflammatory, neuro-protective and lipid peroxidation deterrent activities in AD treatments via different gene regulatory/suppressive mechanisms [189, 216].

#### Parkinson’s disease

Parkinson’s disease (PD), is the second most common progressive multisystem neuro-degenerative disorder and probably occur in aged people. Particularly affects motor and non-motor functions due to loss of dopaminergic neurons. Oxidative stress and neuro-inflammations are the major foundations of PD progressions. Assured treatments using keto-carotenoids especially AST, were found as an effective bioactive for prevention of PD progression and/or restoring the loss of neurons [189].

Successive four weeks supplementation of *Haematococcus pluvialis* derived AST in PD mice have shown reduced neurotoxicity [217]. Grimmig and collaborators have established an anti-inflammatory effects of AST, showing reduced microglial activation in striatum and substantia nigra regions of basal ganglia. The sustained microglial activation leads to accumulation of pro-inflammatory molecules, which will be harmful to the neural environment dure to their cytotoxic natures [217]. In other studies, dose-dependent antioxidant effects of AST were revealed, where ROS mediated apoptosis was found to be declined in dopaminergic SH-SY5Y cell lines [218]. AST has repressed the incidences of DHA hydroperoxide (DHA-OOH) or 6-hydroxydopamine (6-OHDA) stimulated apoptosis cascade, mitochondrial defects and ROS generations [219, 220]. Ikeda Y. showed AST treatment shutdowns 6-OHDA tempted apoptosis and mitochondrial dysfunction via p38 MAPK phosphorylation blocking and condensing caspase 3/9 and poly (ADP-ribose) polymerase action [189, 219]. Recently AST has been reported for Ca^2+^ ion transportation in the brain and also in controlling proper signalling activities [185–187].

In a rat model of homocysteine (Hcy) induced hippocampal neurotoxicity and apoptosis, the AST was found to control oxidative stress-induced damages and mitochondrial dysfunctions. It also diminished PI3K/AKT and mitogen-activated protein kinase (MAPK) pathways, which are important in regulating cell growth or cell deaths. That has enabled a blocking of PD and other neurological disorders so far [221]. In catecholamine type of cells such as PC12, AST was found to suppress the oxidative stress induced by MPP^+^ via SP1/NR1 and HO-1/NOX2 pathways [222, 223].

#### Ocular diseases

Optic neuropathy is an injury to the optic nerves leading to vision losses. It occurs either due to demyelination, inflammation, ischemic or traumatic causes. The glaucoma is a one of the progressive optic neuropathies causes due to damage in the nerve fiber layer and is the second leading cause of blindness worldwide. Cort and co-authors have observed that the glaucomatous retinal injury in mouse can be recovered after treatment with AST [224]. In nitroglycerin (NTG)-induced migraine mouse models, the AST has shown neuroprotective properties. In case of multifactorial disease i.e., glaucoma; AST can also become a potent remedy for its therapeutic treatment [225].

Lin and co-authors have examined the neuroprotective effects of AST in ischemic optic neuropathy mice model. At molecular level, it reduced the expression of TNFα and IL1β in eye retinas, while oxidative and inflammatory mechanisms have also got down-regulated [226]. In another study, an oxidant effect showed suppression in ischemic caused retinal cell death [225, 227].

The studies of Giannaccare et al. have shown the efficacy of AST in the prevention and treatment of various ophthalmic diseases and disorders including retinal diseases, uveitis, asthenopia (eye fatigue), cataract and ocular surface disorders. Earlier reports on animal and human ocular diseases highlighted that the use of AST retains cellular homeostasis by reducing oxidative stress and controlling different metabolic activities, etc. Several clinical trials needed to be performed for getting an idea about dosage quantities, route and duration of administration of the AST to tackle with various ocular damages, diseases as well as disorders [56].

#### Carpel tunnel syndrome

Carpel tunnel syndrome (CTS) is broadly characterized as the compression neuropathy [228]. Non-operative management [229, 230] and conventional medications such as injections, non-steroidal anti-inflammatory drugs and recuperation therapies (ultra-sound, stretching, and strengthening) have been evaluated against CTS [231–233].

Some factors like correct nutritional supplementations could help positively in the CTS managements [234–236]. AST was investigated for its effectiveness on the splinting management in CTS. The electromagnetically confirmed CTS experimental group was supplemented with 4 mg AST (12 weeks) and compared to the control (placebo) group. Both the groups were observed to reduce symptoms which are measured through Symptom Severity Scale (SSS). The arm, shoulder and hand disabilities survey didn’t show significant effects. However, impairment measures showed marginal changes in grip, agility and sensations [237]. So there must be a scope for the further studies to understand and optimize probable usage of AST in CTS management.

### Stress, fatigue and depression

Oxidative stress, psychological stress and fatigue are interconnected processes in life. Exhaustive mental and physical daily routines induce more oxidative stress. Phosphatidylcholine hydroperoxide (PCOOH), is an oxidative stress marker produced after phospholipid oxidation [193]. AST has the ability to suppress the PCOOH levels, which eventually decreases the chances of people suffering from chronic fatigue syndrome. Also, AST has been reported to heighten the strength [238] and fat burns during workout by CPT-I activation [157]. Imai A. et al. study has shown the anti-fatigue effects of AST and its role in the reduction in oxidative stress markers in healthy people performing their daily mental and physical routine [239].

According to Kim et al. report, AST effectively lowered oxidative stress produced during various pathological environments. It maintains structural and functional activities of mitochondria and also prevents the progression of diseases like cardiovascular, neurodegenerative, liver-related malfunctions such as NAFLD, diabetes, hyperglycemia and other secondary complications. In short, AST rich diet or supplementation possibly provides a prevention against ageing processes and health-related problems [240].

Hayashi et al. investigated the relative effect of nutritional supplement of AST (from *Paracoccus carotinifaciens*) on stress and sleep behaviors among 20–60 age group of people. After providing daily oral intake of 12 mg AST for successive 8 weeks, the efficacy of treatment was measured in terms of a primary and secondary outcome as POMS (mental stress) and OSA-MA (sleep) respectively. A change in mood swings and depression-dejection states were found to be decreased and quality sleep act was improved in AST ingested group than placebo group with no adverse effects [241].

### Fertility

The fertility is one of the major concern of health managements in such hectic times. The problem of infertility is getting worsen day by day. The probable reason behind that is the stressful lifestyles and non-healthy diets. Therefore, it was believed that the dietary supplementation might re-regularize the disturbed fertility cycles.

#### Male fertility

A dietary supplementation of AST in combination with vitamin E, vitamin C, and calorie-restricted diet in male rats, has shown to increase count and quality of the sperms. It was also reported to improve sperm capacitation and related functions by augmenting redox system balance in sertoli cells [242, 243]. Recently, Wang et al. stated protective action by AST in steroidogenesis from H_2_O_2_ induced ROS in mouse Leydig cells [244]. In another study, decreased leptin levels showed increased sperm count and motility and thus elevated events of male fertility [245].

According to Donà et al. in-vitro studies, AST could enter in acrosome membrane of sperm and it assists protein phosphorylation (Tyr-p) in the sperm head without inducing ROS (H_2_O_2_) levels. Ultimately enhances the capacitation process by which an easy acrosome reaction (AR) occurs. It also triggers the release of acrosin hydrolytic enzymes which permit sperm and oocyte fertilization [246].

In another reported clinical trial, infertile males with oligoasthenozoospermia were orally supplemented with 4 mg AST and 10 mg vitamin E combination. The semen quality, fertilization and embryo development processes were investigated with their female partners by the assisted reproduction techniques (ART) such as intracytoplasmic sperm injection (ISCI) [247].

Conclusively, we can have a hopeful approach in male fertility problems with an effective supplementation of AST to the patients.

#### Female fertility

In animal studies, AST feeding in chickens has improved egg quality (shelf life) and the colour of egg yolk. However, in pigs’ carcass characters and meat quality was found to be increased [248]. In case of AST fed minks, the amplified quantity of corpora lutea (CL) along with increased implantation sites and fetus numbers was observed. However, stillborn kits number was observed to be decreased [249].

In another study, the effect of AST during in vitro growth (IVG) was inspected. In this, processes such as oocytes competence development and steroidogenesis of granulosa cells devised from early antral follicles were studied. Due to the antioxidant potential of AST, increased total biomass in blastocysts was found. When supplemented in IVG medium, it amended the overall quality and growth of bovine oocytes, also suppressed granulosa cells’ luteinisation [250].

In postmenopausal women studies, AST promoted vascular endurance and increased antioxidant capacities by reducing elevated levels of oxidative stress. Successive 8-week intake of 12 mg AST in diet significantly reduced systolic and diastolic blood pressure. A drop in vascular resistance in the lower limbs and serum adiponectin, improved physical and mental symptoms were also noted [10, 251].

Recently some researchers have also performed clinical trials concerning the effect of AST on oxidative stress management in women suffering from polycystic ovary syndrome (PCOS) [252]. The AST administration was expected to reduce ROS in FF and induction of antioxidant response elements in PCOS women.

### Anti-tuberculosis

Mycobacterial infections and multidrug-resistant (MDR) strains of *Mycobacterium tuberculosis* (MTB) are one of the foremost causes of mortality and a major reason for socioeconomic burden worldwide [253]. Yearly, about 8 million new tuberculosis cases and 2 million deaths were reported [254]. The number might have increased now. According to a WHO survey, about one-third of the global population is at risk of MTB infections [255].

Recently, the anti-mycobacterial activity of AST and liposome-encapsulated AST were explored. The docking studies of AST with different mycobacterial drug target proteins have provided good interactions than the conventional anti-TB drugs i.e. isoniazid and rifampicin. However, an encapsulated AST displayed better inhibition of mycobacterium (MIC value of 500 μg/mL) against MDR-TB, and H37Rv. Future studies on animal models could provide insight for identifying the exact mechanism of action of AST in TB managements [256].

### Anti-viral

Recently Donà et al. appraised the protective effects of AST on isolated and purified human sperm in the presence of human papillomavirus (HPV) 16 capsid protein, L1. AST had shown strong antiviral effects by preventing the binding of HPV L1 to the sperm membrane and thus HPV entry was prohibited [257]. The study may encourage to evaluate, more antiviral capabilities of AST with various viruses.

### Anti-COVID 19

In the COVID-19 disease, the inflammations caused by a novel species of SARS CoV-2 and elevated levels of cytokine storms as well as inflammatory factors such as interleukins (IL-1β, IL-6, IL-8), tumor necrosis factor (TNF-α), etc. can be regulated and suppressed by the supplementation of AST. So it can be a promising therapeutic dietary supplement along with primary antiviral medications for the patients suffering from severe pneumonitis, acute lung injury (ALI), Acute Respiratory Distress Syndrome (ARDS), other lungs related infections and also in recovered patients of COVID-19 as a post-treatment care [258–260].

The AST was observed to decrease membrane fluidity and raises the activation of nuclear factor erythroid 2–related factor 2/heme oxygenase-1 pathway. The pathway further stimulates an increased synthesis of antioxidant enzymes viz., peroxidase, catalase, superoxide dismutase, NAD(P)H quinine oxidoreductase-1, glutathione-S-transferase-α1, and thiobarbituric acid reactive substances [261]. AST was also found as a potent inhibitor of superoxide radicals, ROS, cytosolic calcium, myeloperoxidase (MPO), and other oxidative mediators and lipid peroxidation as well. The acceptable dietary intake of AST for COVID-19 is of 2 mg/day, which has been accepted in European Food and Safety Authority and about 24 mg/day in the US [262]. In the US, their FDA has approved a “no objection” certificate for about 20 new dietary components including AST with daily uptake in between 2 mg and 24 mg [263]. In this way the AST and its preparations are getting popular and being acceptable worldwide in the COVID-19 management. However, the effectiveness and exact efficacy of it is yet to be determined at molecular level.

## Nutraceutical potential of astaxanthin

In aquaculture and food industries, AST is mainly used as an additive and dietary supplement. Among several carotenoids, β-Carotene and AST are typical components used in chicken and fish feeding, respectively [264]. AST enhances aesthetic qualities as well as the market value of sea-foods like salmons, shrimps, trouts, lobsters, crayfish, fish eggs and also the ornamental fish [38, 43, 101, 265–267]. In farmed Atlantic salmon AST content was reported up to 6 mg/kg–8 mg/kg flesh. In the large trout from European and Japanese markets, 6 mg/kg flesh and 25 mg/kg flesh of AST content was observed respectively [177]. In nature, *Haematococcus pluvialis* derived all *trans* 3*S*,3′*S* AST is the utmost reported source so ever [43].

In 1987, USFDA authorized AST as a feed supplement in aquaculture industries, and in 1999, AST again approved for its use in food industries too [101]. Globally emerging aquacultures, food industries and their massive demands have fortified AST’s commercial value; Japan is one of the topmost pioneers in AST production and research for those applicatory usages [105].

Several clinical studies have revealed AST’s remarkable anti-oxidative effects, multifunctional health benefits, and its bio-safety properties. Hence, it is now also being used in the cosmetics and nutraceutical industries too. Some globally recognized manufacturing brands producing AST (as a nutraceutical) are listed in Table 2. In some countries, the AST is occasionally used to strengthen the quality of foods and brews [21]. Currently, > 95% of the AST production in the market has been occupied by synthetic AST [38]. However, the natural AST has a great consumer demand and therefore it is used in food, cosmetics and nutraceutical industries. In a global scenario, the AST market is estimated to reach up to 3398.8 million by the year 2027 due to its well-rising alertness in the health and nutrition sector [21]. Assuredly, in emerging decades, valuable AST and its promising bioactivities will endorse its up-surging potency for the betterment of human health.

**Table 2:** Astaxanthin (as a nutraceutical) manufacturing global brands (from different biological sources)

Sr. No	Product Name	Brand Name
Source: *Haematococcus pluvialis*		
1	ASTAREAL®	BodyFirst^®^
2	Astaxanthin	Zenith Nutrition
3	Astaxanthin	Healthy Hey
4	Astaxanthin	Himalayan Organics
5	Astaxanthin	Sports Research
6	Astaxanthin	Nature’s Velvet
7	Astaxanthin	Now Foods
8	BTN Super Antioxidant Astaxanthin	Bionova Targeted Nutrition
9	Astaxanthin	Truebasics
10	Astaxanthin	Doctor’s Best
11	BioAstin^®^ OmegaAstin™	Hawaiian Herbal
12	Astaxanthin with Phospholipids	Life Extension^®^
13	Astaxanthin, AstaLif^®^ Pure Icelandic	California Gold Nutrition
14	SuperPure^®^ Astaxanthin	Pure Synergy
15	Astashyn™	Newtrimed Healthcare Private Limited
16	Astaxanthin	TRUHEALTHY^®^
17	Oriflame Wellness Astaxanthin And Bilberry Extract	Oriflame
18	Astaxanthin	Source Naturals^®^
19	Astaxanthin Plus	Natural Factors^®^
20	Natural Astaxanthin	MicrOrganics Green Nutritionals
21	Natural Astaxanthin Triple Strength	Healthy Origins^®^
22	Astaxanthin	Ramini^®^ Bio Nutrition
23	Astaxanthin	Sam Health^®^
24	Natural Astaxanthin	Solgar^®^
25	Vegan Astaxanthin	Deva^®^
26	Natural Astaxanthin	Vaddmaan
27	Astaxanthin	Jarrow Formulas^®^
28	Astaxanthin	We Like Vitamins
29	High-Potency Astaxanthin	Swanson^®^
30	Astaxanthin	Viva Naturals
31	Astaxanthin	Simply Potent
32	Astaxanthin	aSquared Nutrition
33	Astaxanthin Royal	Vitality Nutritionals
34	Astaxanthin	ICEHERBS Natural Supplements
35	Astaxanthin	Webber Naturals
36	Astaxanthin	Neurapid
37	Astaxanthin Oil	Horbäach^®^
38	Xantox™ VF	Glenmark Pharmaceuticals
39	Astaxanthin	Cheeky Nutrition
40	Natural Astaxanthin	MicrOrganics
41	Astaxanthin	Terranova
42	AstaFit^®^	BioLife Science^®^
43	AstaXanthin™ with DHA	Good Health Naturally Nutrition™
44	Sila Astaxanthin	LJS International Development Co., Ltd
45	AstaFX^®^ Astaxanthin Super Formula	Purity Products
46	AstaVibrance Astaxanthin	BioPure.eu
47	TrueAsta™	Nature City^®^
48	Astaxanthin (New)	NutriGuard™
49	Astaxanthin	Design for Health^®^
50	Astaxanthin	Prime™
51	ASTASHINE™	Fuji Chemical
52	Nature Astaxanthin	Newmstar
53	Astaxanthin Forte	OstroVit^®^
54	Astaxanthin	Bio Natura
55	Astaxanthin Beadlets	Fairvital
56	Astaxanthin	Biosphere
57	Astaxanthin	Amazing Nutrition
58	Astaxanthin	Waka Tani
59	Astaxanthin	Solaray^®^
60	K2-D3 with Astaxanthin	Douglas Labs
61	Astaxanthin Vegan Max	Barlowe’s Herbal Elixirs
62	Astaxanthin	California Products
63	Astaxanthin Extract	HerbaDiet
64	Astaxanthin	BioBalance^®^
65	AstaReal^®^ Astaxanthin	AstaVita^®^
66	Astaxanthin	Viridian
67	Astaxanthin Plus	Prairie Naturals^®^
68	Astaxanthin	Vibraxlabs
69	Astaxanthin	Getilapson™
70	AstaReal^®^ Oil (Oleoresin) AstaReal^®^ Powder AstaReal^®^Cold Water Soluble Powder AstaReal^®^Water Soluble Emulsion AstaReal^®^Bulk softgel capsules AstaMed MYO™	AstaReal^®^ AstaMed^®^
71	Astaxanthin	ALG*O*RIGIN
72	Natural Astaxanthin	Vitacost
73	Astaxanthin	Kala Health
74	Astaxanthin	BIOMED_TM_
75	Astaxanthin	Health Supplements
76	GO Astaxanthin Antioxidant High Strength	GO^®^ healthy
77	Natural Astaxanthin	PipingRock^®^
78	AstaZine^®^ Astaxanthin	NutriCology^®^
79	Astaxanthin	Healthy Directions
80	Organic Astaxanthin	EyeScience^®^
81	AstaREAL^®^ Astaxanthin	Nature’s Lab
82	Astaxanthin	Trunature^®^
83	Natural Astaxanthin	Puritan’s Pride
84	Astaxanthin	Dr. Mercola^®^
85	Vision Advantage^®^	Dr. David Williams
86	Astaxanthin	Dr. Whitaker
87	Astaxanthin	Lamberts^®^
88	AstaZan™	LifeStream^®^
89	Astaxanthin	The Vitamin Shoppe^®^
90	Astaxanthin 12,000	Andrew Lessman’s Procaps Laboratories
91	Astaxanthin Gold	NutriGold^®^
92	Astaxanthin	Time Health
93	Astaxanthina	Yamamoto^®^ Research
94	Asta-i-Shield	Vitamode^®^
95	Astaxanthin	Regenurex™
96	Organic Astaxanthin	Naomi
Source: *Phaffia rhodozyma*		
97	Astaxanthin Gummies with Vitamin C (Astaferm™)	Carlson Labs
Source: Antarctic Krill (*Euphausia superba*)		
98	High-Potency Krill Oil With Astaxanthin	WholeHealth^®^
99	Vestige Krill Oil	Vestige
Source: Unknown		
100	Astaxanthin Super Antioxidant	Simply Herbal
101	Astaxanthin Natural	Woohoo Natural^®^
102	Eye Health Support	Allbeing
103	Astaxanthin	Naturalis
104	Astagold	Sunways (India) Pvt. Ltd
105	Astaxanthin	Ayurveda Redefined
106	Astaxanthin	HealthVit
107	Astaxanthin	Higher Nature^®^
108	Avanza^®^ Astaxanthin	Healthcare Pharmaceuticals Limited
109	Astaxanthin	NUHEALTH^®^

In this way, the astaxanthin has been involved and being considered in many important applications as cosmetics, food and feed industries. And now it is getting evolved as the key ingredient of certain nutraceuticals, due to its potent antioxidant activity. Which is > 10 times efficient than the β-carotene and about 1000 times more effective than the vitamin E. The multiple potencies of AST, such as protection against UV-radiation, photo oxidation, inflammation, cancer, ulcer, *Helicobacter pylori* infection, aging, and age-related ailments, or in the promotion of the immune response, liver functions, heart, eye, joints and prostate health, etc. have elevated its importance as the crucial substance in the formulations and developments of many nutraceutical products [268].

## Biosafety and bioavailability of astaxanthin

Earlier studies say that AST is absolutely safe for consumption when taken with food or as a dietary supplement. The studies on rat models revealed that AST accrues in animal tissues without any lethal effects [269–271]. But yes, if excessive AST get consumed it leads to yellow to reddish pigmentation of the skin in animals. For example, AST incorporated into fish feed, causes reddish skin colour in fish. Along with lipid-based formulations, AST’s bioavailability can be amplified [269, 270, 272]. It was found that, super therapeutic concentrations of AST had led no antagonistic effects on platelet, coagulation and fibrinolytic functions [273]. Considerably, no research has reported on significant side effects of AST intake in animals as well as in humans. These results provide backing up for the safety of AST for future clinical studies.

According to some studies, it is advised that AST consumption with omega-3 rich seed oils such as flaxseed, chia, fish, nutella, almonds and walnuts could enhance its bioavailability. The blend of AST (4 mg–8 mg) with foods, capsules, soft gels and skin creams is available in the market. The suggested dose of AST is 2 mg/day–4 mg/day for adult humans. Even no harmful effects were seen at its slightly increased the dose (6 mg/day) [177, 274].

## Conclusions and forthcoming directions

The awareness and yearning among the people about ‘wellness industry’ than the earlier ‘illness industry’ is increasing day by day. Which will work as a driving force for development of health supplementary products based on natural healing and nurturing mechanism. The AST, a versatile natural bioactive pigment has been acknowledged by the global scientific society in concern with its health benefits of fulfilling the nutritional gaps of continued increasing populations. This also underlines its importance in the nutraceutical supplementations as well as in the prevention and protection strategies against many pathological problems. The AST is a great antioxidant having about 110 times more anti-oxidation potency than the Vitamin E, 560 times than green tea catechin, 800 times than Coenzyme Q10, 3000 times than resveratrol and about 6000 times more than vitamin C. Also the superiorities of AST such as the potent anti-oxidant, anti-inflammatory, anti-proliferative, anti-cancer, anti-obese, anti-diabetic, anti-ageing, anti-TB, anti-viral, anti-COVID 19, neuro-protective, nephro-protective, and fertility-enhancing properties make it is the most precious thing to be studied for prevention as well as for the treatment of prevalent systematic diseases and/or disorders. Although several reports strongly support the safety profile of AST in human uses, several in-vivo and in-vitro studies have been reported for its nutritional, therapeutic, supportive, regulatory, modulatory, protective and preventive actions. However, a deep investigation is need to be performed in various physiological diseases and disorders. That will clarify its exact mechanism of actions in control and cures at the molecular levels of the cells. While the analysed data in the current review studies suggests that the AST possesses an incredible pharmaceutical, nutraceutical and bio-functional properties. It is safe for human use and has a great bio-acceptability too. But since it cannot be synthesized by animals, it has to be provided them as their dietary supplement to maintain a balanced natural living.

The current review also summarises the primary as well as secondary natural sources of AST, various factors affecting its biosynthesis, followed by its potential applications in pharmaceutical formulations. The superlative and incredible usage of AST in treatment of obesity issues, CVDs and its allied complications, cancer, respiratory as well as liver diseases, stress, fatigue, depressions, aging and fertility issues, neurological and neuro-degenerative diseases, autoimmune disorders, immune-pathogenicity and immune-modulatory functions have been comprehensively discussed and documented in the report. The functional usages of AST in manipulation of tuberculosis, viral infections and in COVID-19 disease have also been highlighted here.

At last, the review report is also detailing the existing as well as the prospective applications of AST in nutraceutical industries. The increments in nutritional as well as aesthetic qualities of sea foods with reference to the European and Japanese market have been discussed here. The review also encompasses the data of biosafety as well as the bioavailability of AST. Its prospected universal market potential and estimated market reach by coming decades is also elaborated further.

In conclusion, the study is going to add some important understandings in the global knowledge base of the field and it may further offer a great chunk of opportunities in coming years. It also provide a promising hope that the AST and its bioactive potential with respect to the pharmaceutical and nutraceutical approaches will uplift the human health and it may further guide the people to battle wisely against many life-threatening health concerns. The review may also encourage few new groups of researchers to explore its futuristic nutraceutical ventures. That is anticipated because the demand and acceptance of the nutraceuticals are going to be one of the prime obligation of human beings in future.
