# Editorial Expression of Concern: Pharmaceutical and nutraceutical potential of natural bioactive pigment: astaxanthin

**DOI:** 10.1007/s13659-023-00370-7

**Published:** 2023-02-01

**Authors:** Apurva D. Patil, Pramod J. Kasabe, Padma B. Dandge

**Affiliations:** 1 Department of Biochemistry, Shivaji University, Kolhapur, 416004 Maharashtra India; 2 School of Nanoscience and Biotechnology, Shivaji University, Kolhapur, Maharashtra India

**Editorial Expression of Concern: Natural Products and Bioprospecting (2022) 12:25** **https://doi.org/10.1007/s13659-022-00347-y**

After publication of the original article [1], the Editor and Publisher became aware that the authors did not have permission to reproduce the image in Fig. 1, “Orientation of astaxanthin, β-carotene, vitamin C and vitamin E in plasma membrane”. Furthermore, the authors did not provide evidence of obtaining permission to use the images comprising of Fig. 3 “Astaxanthin applications in various industries." As a result, Figs. 1 and 3 have been obscured in the original article for legal reasons.

## References

[1] Patil AD, Kasabe PJ, Dandge PB (2022). Pharmaceutical and nutraceutical potential of natural bioactive pigment: astaxanthin. Nat Prod Bioprospect.

